# Metformin: A Candidate Drug for Renal Diseases

**DOI:** 10.3390/ijms20010042

**Published:** 2018-12-21

**Authors:** Raphaëlle Corremans, Benjamin A. Vervaet, Patrick C. D’Haese, Ellen Neven, Anja Verhulst

**Affiliations:** Laboratory of Pathophysiology, Department of Biomedical Sciences, University of Antwerp, 2000 Antwerp, Belgium; raphaelle.corremans@uantwerpen.be (R.C.); benjamin.vervaet@uantwerpen.be (B.A.V.); patrick.dhaese@uantwerpen.be (P.C.D.); ellen.neven@uantwerpen.be (E.N.)

**Keywords:** metformin, type 2 diabetes mellitus, acute kidney injury, chronic kidney disease, lactic acidosis, renoprotection, AMP-activated protein kinase pathway

## Abstract

Over the past decades metformin has been the optimal first-line treatment for type 2 diabetes mellitus (T2DM). Only in the last few years, it has become increasingly clear that metformin exerts benign pleiotropic actions beyond its prescribed use and ongoing investigations focus on a putative beneficial impact of metformin on the kidney. Both acute kidney injury (AKI) and chronic kidney disease (CKD), two major renal health issues, often result in the need for renal replacement therapy (dialysis or transplantation) with a high socio-economic impact for the patients. Unfortunately, to date, effective treatment directly targeting the kidney is lacking. Metformin has been shown to exert beneficial effects on the kidney in various clinical trials and experimental studies performed in divergent rodent models representing different types of renal diseases going from AKI to CKD. Despite growing evidence on metformin as a candidate drug for renal diseases, in-depth research is imperative to unravel the molecular signaling pathways responsible for metformin’s renoprotective actions. This review will discuss the current state-of-the-art literature on clinical and preclinical data, and put forward potential cellular mechanisms and molecular pathways by which metformin ameliorates AKI/CKD.

## 1. Introduction

Metformin is widely accepted as the first-line therapy of type 2 diabetes mellitus (T2DM), because of its capacity to lower blood glucose in association with beneficial effects on plasma lipids, body weight, and a low incidence of micro- and macrovascular events [1,2]. It is one of the oldest and most prescribed antidiabetic drugs worldwide [3]. Historically, metformin originates from galegine, a guanidine derivative found in *Galega officinalis*, and was used in herbal medicine in medieval Europe [2,4]. After the discovery of its glucose-lowering properties in the 1920s, metformin has gone through a turbulent route of being disregarded, forgotten, rediscovered, and repurposed. It was ultimately Jean Sterne who first reported the use of metformin, introduced as “Glucophage”, to treat diabetes in 1957 [5,6].

Metformin acts primarily in the liver, where its uptake is mediated by organic cation transporter 1 (OCT1), by inhibiting hepatic gluconeogenesis and glycogenolysis [1,4]. It enhances peripheral glucose uptake and utilization, mainly in the muscles, by improving insulin sensitivity. Further, an important contribution to the antihyperglycemic efficacy of metformin arises from actions within the gut, by increasing glucagon-like peptide 1 (GLP-1) secretion, and possibly altering the gut microbiome [3,4,7,8]. GLP-1, a glucose-lowering gut incretin hormone, secreted in response to food ingestion, stimulates glucose-dependent insulin secretion, and inhibits glucagon release from the endocrine pancreas, which normalizes glycaemia [9]. In addition, GLP-1 slows gastric emptying and, thus, reduces appetite and food intake, which is essential for postprandial glucose control [9]. Furthermore, metformin interacts with different gut bacteria, possibly through the regulation of metal homeostasis, which might also contribute to the glucose-lowering effect of metformin [10]. Metformin is not generally associated with a risk of hypoglycemia, because it does not stimulate endogenous insulin production, in contrast to other antidiabetic drugs [3,7].

In the last decade, the use of metformin in T2DM, to simply lower blood glucose, has switched to a much more complex picture, due to its benign pleiotropic effects. Metformin has shown substantial effectiveness in polycystic ovarian syndrome [11], cancer [12], heart and cardiovascular disorders [13], nonalcoholic fatty liver disease [14], and premature puberty [3,15]. Furthermore, ongoing investigations focus on its putative beneficial impact on the kidney. Metformin, when administered during acute kidney injury (AKI) in rats, has been demonstrated to attenuate ensuing chronic renal impairment [16], whilst pretreatment of healthy kidneys significantly protected the kidney from subsequent AKI [16,17,18]. Additionally, metformin has shown a beneficial and strong protective effect against the progression of chronic kidney disease (CKD) [19]. The aim of this review is to summarize the current knowledge regarding the renoprotective effect of metformin. We will review the available clinical and preclinical evidence that points towards a protective effect of metformin treatment on the development/progression of renal diseases with different underlying etiologies, and will discuss the cellular mechanisms and potential molecular signaling pathways by which metformin exerts its beneficial impact on the kidney.

## 2. Metformin’s Glucose Lowering Effect: Molecular Mechanisms

Despite its clinical use and detailed investigations for more than 60 years, the molecular pathways by which metformin lowers blood glucose are still not fully understood [4,7]. A decade ago, it was generally accepted that metformin reduced hepatic glucose synthesis via activation of AMP-activated protein kinase (AMPK) through decreases in hepatic energy charge or directly by its upstream activator liver kinase B1 (LKB1). Hepatic gluconeogenesis is an energy-intensive process which starts in the mitochondria, where pyruvate enters the gluconeogenic route [20]. Metformin (>1 mM in vitro) is able to inhibit complex 1 of the mitochondrial respiratory chain, which suppresses ATP production [4,21,22]. However, it needs to be mentioned that the claimed effect of metformin should be interpreted with caution since several in vitro studies were conducted with supra-pharmacological concentrations. After oral administration of metformin, a plasma concentration of 10–40 μM is achieved in humans [23,24]. Subsequently, cytoplasmic ADP/ATP and AMP/ATP ratio’s will increase and activate AMPK, leading to reduction of gluconeogenic gene transcription [4,21,25,26]. Interestingly, recent studies report that metformin inhibits gluconeogenesis through AMPK-independent mechanisms. Foretz et al. [27] demonstrated that metformin (>250 μM in vitro) inhibited hepatic gluconeogenesis via a decrease in the hepatic energy state in an AMPK-independent manner. They used genetic ablation of both AMPK catalytic subunits and LKB1, to show that both AMPK and LKB1 are not required for metformin-induced (50 mg/kg, 150 mg/kg, 300 mg/kg p.o.) decreases in hepatic glucose output in mice. They found that in mice lacking AMPK in the liver, blood glucose levels were comparable to those in wild-type mice, and that the hypoglycemic effect of metformin was maintained [27]. Counteracting hepatic glucagon signaling by metformin, as a novel contributor to its therapeutic actions, was reported by Miller et al. [28]. In this study, metformin treatment (250 mg/kg/day p.o.) reduced levels of cyclic AMP, as a result of adenylate cyclase inhibition and, consequently, attenuated glucagon’s ability to promote hepatic glucose production [28]. Later, Madiraju et al. [29] showed that metformin (50 μM in vitro, single dose 50 mg/kg i.v.) inhibited mitochondrial glycerophosphate dehydrogenase, resulting in an altered hepatocellular redox state, ultimately leading to impaired utilization of lactate and glycerol for gluconeogenesis [29]. Recently, a new mechanism of action was discovered by Hunter et al. [30]. Metformin (250 mg/kg single dose p.o.) inhibited fructose-1-6-bisphosphatase allosterically, by increasing AMP concentration [30]. These findings clearly demonstrate that the underlying mechanisms responsible for the glucose-lowering effects of metformin in diabetes may not be explained by any single target or pathway.

## 3. Acute and Chronic Kidney Disease

Before reviewing the molecular signaling pathways by which metformin has been shown to protect the kidneys, we will briefly elaborate on AKI and CKD. AKI is defined as an abrupt and rapid loss of renal function that occurs rapidly over a few hours or days, and is mainly caused by exposure to nephrotoxic substances, impaired renal blood flow, obstruction of the urinary tract, or inflammation in the kidney [31]. CKD represents a progressive loss of renal function over a period of months or years, often (but not consistently) progressing towards end stage renal disease (ESRD), which inevitably requires renal replacement therapy, i.e., dialysis or kidney transplantation. However, distinction between AKI and CKD may be artificial, since both syndromes are closely interconnected [32].

### 3.1. Acute Kidney Injury

AKI is an increasingly common complication in, often elderly, patients admitted to hospitals [33]. AKI is defined as an increase in serum creatinine of more than 0.3 mg/dL within 48 h, or an increase in serum creatinine to 1.5 times baseline, which is known, or presumed, to have occurred within the prior 7 days, or a urine output of less than 0.5 mL/kg/h for 6 h [34]. Clinically, AKI compromises three primary etiologies, including prerenal (azotemia), renal (tubular necrosis, interstitial nephritis or acute glomerulonephritis), and post-renal (acute obstruction to urinary flow) [35,36]. Hypoperfusion, a complication of major surgery and sepsis, can induce ischemia-reperfusion injury to the kidney and, together with exposure to nephrotoxins, is the main cause of AKI [37,38]. Patients need to be managed according to the cause of their renal disease, although the wide variety of injuries that can occur to the kidney makes the latter challenging. Furthermore, despite the regenerative capacity of the kidney to restore its function, patients who survive AKI have an increased risk of developing CKD and ESRD [39].

### 3.2. Chronic Kidney Disease

**Figure 1:**
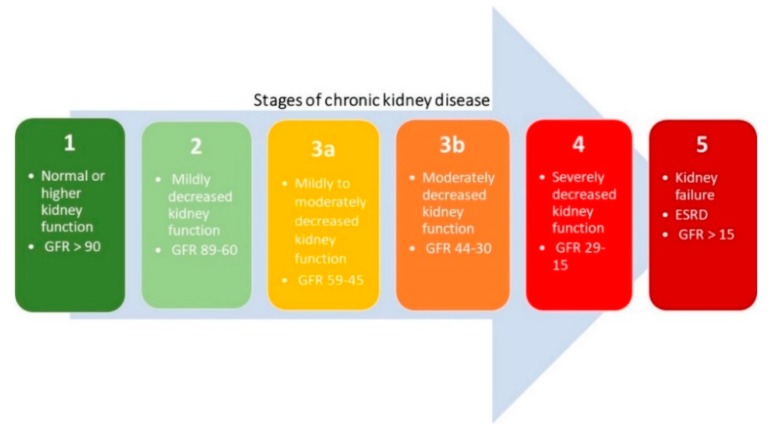
Five stages of chronic kidney disease based on the estimated glomerular filtration rate (eGFR) (mL/min per 1.73 m^2^).

CKD is a worldwide recognized public health issue affecting 3–16% of the world population [40,41]. CKD is defined as histopathological kidney damage and/or decreased kidney function, lasting more than 3 months [42]. Kidney damage usually precedes alterations in function [43]. The kidney structure is considered to be affected when one or more markers of kidney damage are present, such as proteinuria and/or abnormalities detected by histology or non-invasive imaging [42,43,44]. The histopathological features of CKD are tubulointerstitial fibrosis, tubular atrophy, cellular infiltration, and glomerulosclerosis [45]. The most prominent functional parameter of the kidney is the glomerular filtration rate (GFR), which equals the total amount of fluid filtered through the glomeruli per unit of time [44,46]. The Kidney Disease Outcomes Quality Initiative (KDOQI) of the National Kidney Foundation (NKF) divided CKD into five stages, regardless of underlying causes, which are shown in Figure 1. A decreased estimated glomerular filtration rate (eGFR) of less than 60 mL/min/1.73 m^2^ is defined by the current international guidelines as CKD [42,47]. When the eGFR is less than 15 mL/min/1.73 m^2^, the final stage of renal failure, at which kidney function is no longer sufficient to meet the body’s need, is reached [42,44].

In developed countries, age, diabetes, hypertension, cardiovascular disease and obesity, are the leading causes of CKD [44]. These traditional CKD risk factors are accompanied by nontraditional CKD risk factors, such as infections, kidney stones, and exposure to drugs and toxins, which result in glomerular and tubulointerstitial diseases that contribute to the global burden of CKD [40,44,48].

### 3.3. Treatment

Current treatment strategies for AKI are mainly supportive with the correction of volume overload and biochemical abnormalities as primary goal of treatment [31]. Also, for CKD, treatment strategies mainly focus on controlling the above-mentioned risk factors by administration of conventional medication. However, both the IDNT (Irbesartan Diabetic Nephropathy Trial) and RENAAL (Reduction of Endpoints in Non-insulin-dependent T2DM with the Angiotensin II Antagonist Losartan) studies have shown that patients progressively lose kidney function whilst being treated with these medications [49,50]. To date, effective treatment directly targeting the kidney to halt progression of CKD, to attenuate AKI, or expedite recovery, is lacking. In view of the expanding AKI/CKD population in our aging society, the demand for new therapies with far-reaching clinical and social benefits is high. Given the current experimental and clinical data, metformin is an interesting candidate that certainly deserves further research attention.

## 4. Lactic Acidosis during Metformin Treatment in Renal Failure: A Manageable Contraindication

**Figure 2:**
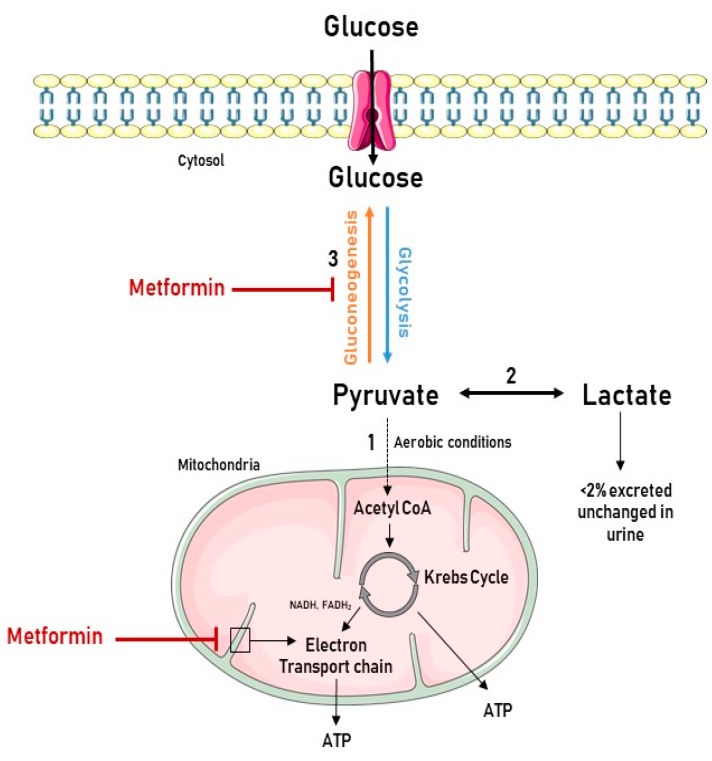
Association between the biochemistry of lactate production and metformin use. Glycolysis is the metabolic pathway that converts glucose into pyruvate in the cytoplasm. Pyruvate is the only precursor of lactate. (**1**) When oxygen is available, pyruvate enters the mitochondria, and is converted to Acetyl CoA and oxidized in the Krebs cycle, delivering nicotinamide adenine dinucleotide (NADH), flavin adenine dinucleotide (FADH2), and adenosine triphosphate (ATP). NADH and FADH2 feed the electron transport chain in the inner mitochondrial membrane to eventually generate the bulk of ATP by chemiosmosis. (**2**) Under anaerobic conditions, pyruvate is reduced to lactate. However, in the liver and kidney, lactate can be converted to pyruvate again by the Cori cycle. (**3**) Pyruvate, the first designated substrate of the gluconeogenic pathway, can be used to generate glucose. Metformin inhibits gluconeogenesis, leading to pyruvate accumulation and subsequent increased lactate production. It also inhibits the electron transport chain, resulting in elevated levels of NADH, a reduced Krebs cycle flow and, hence, further contributing to increased pyruvate levels. Figure was produced using Servier Medical Art: https://smart.servier.com/ (accessed on 25 October 2018).

The historical fear for the development of lactic acidosis has hampered the exploration of the clinical use of metformin in conditions of renal impairment. Lactic acidosis is the most common cause of metabolic acidosis and a well-recognized complication of biguanide therapy [51,52]. It occurs when lactic acid production exceeds lactic acid clearance, and is generally defined as a decreased blood pH (<7.35) accompanied by elevated blood lactate levels (>45.0 mg/dL, >5 mmol/L) [53]. Lactate, formed by the reduction of pyruvate, is produced in the gut, liver, and peripheral tissues as a metabolic end product of anaerobic glycolysis. Under aerobic conditions, pyruvate enters the mitochondria, where its energy is transferred to ATP via the Krebs cycle and oxidative phosphorylation, respectively. During the gluconeogenesis, pyruvate can be converted back to glucose in the liver and kidney [53]. Metformin increases lactate levels by inhibiting complex 1 of the mitochondrial respiratory chain, which negatively impacts (i.e., reduces) the flow through the Krebs cycle as shown in Figure 2. Consequently, metabolic degradation of lactic acid by oxidation or gluconeogenesis is reduced and plasma lactate concentration increases [53,54].

In 1994, the U.S. Food and Drug Administration (FDA) approved the therapeutic use of metformin in T2DM, but contraindicated it in patients with decreased kidney function, as well as in those with liver dysfunction [55,56]. Since metformin is not metabolized, but excreted, unchanged, via urine, the legitimate concern arose that metformin would accumulate in the circulatory system of patients with impaired kidney function, thereby increasing the risk for lactic acidosis [1]. The incidence of “metformin-associated lactic acidosis” (MALA) in T2DM is 3–9 cases per 100,000 patient-years, with a mortality of almost 50% [57,58]. Clinical studies exploring the effect of metformin on the incidence of lactic acidosis are mostly performed in diabetic patients with varying degrees of renal function and report conflicting results. Ekström et al. [59] revealed that use of metformin (±1700 mg monotherapy, >1700 mg combination therapy) was associated with reduced risk of all-cause mortality, acidosis, serious infection, or cardiovascular disease in patients with impaired renal function (eGFR 30 to 45 mL/min per 1.73 m^2^). Richy et al. [60] focused on the risk of lactic acidosis in metformin-treated T2DM patients with various degrees of renal failure. They concluded that metformin use did not affect the incidence of lactic acidosis in patients with T2DM who had either normal, mild, moderate, or severe renal dysfunction [60]. However, Eppenga et al. [61] based on results of a retrospective patient database analysis reported that the risk of lactic acidosis or elevated lactate concentrations is significantly increased in patients with mild to moderate renal insufficiency (<60 mL/min per 1.73 m^2^) in T2DM patients with a recent prescribed dose of >2 g [61]. In 2016, the FDA revised their warning with regard to metformin use in patients with impaired kidney function (eGFR < 60 mL/min/1.73 m^2^), expanding its use in CKD to those with an eGFR of 30 mL/min/1.73 m^2^. The revised guidelines state that metformin could be used safely in patients with mild to moderate impairment in kidney function, but remains absolutely contraindicated in patients with severe CKD (eGFR < 30 mL/min/1.73 m^2^) [62].

Interestingly, a recent clinical study of Lalau et al. [63] indicated that metformin treatment also appeared to be safe and still pharmacologically efficacious in moderate-to-severe CKD, when the dose is adjusted for the degree of renal failure. More precisely, on the basis of dose-finding study’s, they selected a chronic dosage regimen of 1500 mg/day for patients with CKD stage 3a, 1000 mg/day for patients with CKD stage 3b, and 500 mg/day for patients with CKD stage 4 [63].

In conclusion, the historical fear for metformin treatment in patients with renal impairment is seemingly overemphasized. Provided that the dose is adjusted for renal function and a close follow-up of the patients is ensured, metformin can be used safely in patients with mild-to-moderate renal failure. 

## 5. Metformin’s Renoprotection: Clinical Evidence

In several clinical studies, metformin has been shown to improve survival of AKI and CKD patients. In a large cohort of over 25,000 patients with T2DM, Bell et al. [64] provided a reassuring message of the safety of metformin in patients with or without CKD, as mortality was not adversely affected by metformin use. Metformin did not increase the incidence of AKI, and survival rates were higher in patients with AKI previously treated with metformin [64]. In a retrospective cohort study, Stephen et al. [65] linked Scientific Registry of Transplant Recipients data for all incident kidney transplants from 2001 until 2012, and national pharmacy claims. They found that survival was superior for all outcomes for recipients who filled metformin claims compared with those who filled non-metformin agent claims [65]. In an open cohort study of 469,688 T2DM patients, the relationship between a range of complications and antidiabetic therapy was analyzed. Severe kidney failure, including dialysis treatment, kidney transplant, and CKD stage 5, were among the five pre-specified key outcomes. Compared with non-use, metformin was associated with a significantly decreased risk of severe kidney failure, whereas sulfonylureas and insulin increased this risk [66]. In a recent systematic review involving 17 observational studies, metformin use appeared to be associated with reduced all-cause mortality in patients with CKD, congestive heart failure, and chronic liver disease [67].

Conversely, in a study of 616 patients, Hsu et al. [68] evaluated the effect of continuous metformin treatment on renal function in patients with T2DM and moderate CKD (eGFR 30–60 mL/min/1.73 m^2^). They concluded that continuous metformin therapy was associated with a decline in renal function in patients with T2DM and moderate CKD [68]. However, as this was a retrospective study, the authors could not exclude putative confounding factors such as life-style, use of angiotensin-converting enzyme inhibitor/angiotensin receptor blocker, cumulative duration of exposure, and defined daily dose of metformin.

These findings open perspectives for urgently needed large, long-term prospective randomized controlled trials that investigate the effect of metformin on renal function.

## 6. Metformin’s Renoprotection: Experimental Evidence

Due to the fact that metformin has been historically contraindicated in patients with advanced CKD, because of the lingering concern of life-threatening lactic acidosis, a large body of evidence to support the renoprotective effect of metformin is provided by preclinical studies. Metformin has been shown to exert positive effects on the kidney in various experimental studies performed in divergent rodent models representing different types of renal diseases going from AKI to CKD.

Satriano et al. [69] studied the effect of metformin on kidney function and structure in 5/6 th nephrectomized rats, a model of mild-to-moderate CKD, to determine whether changes in AMPK enzymatic activity correlates with the changes in kidney metabolism and kidney function [69]. Daily administration of metformin (250 mg/kg/day p.o.) by oral gavage ameliorated kidney fibrosis, as well as structural and morphologic renal abnormalities. In addition, metformin normalized kidney function and oxygen consumption, which was also mediated via induction of AMPK [69]. Lee et al. [70] evaluated the role of the AMPK-acetyl-CoA carboxylase (ACC) pathway in the antifibrotic effect of metformin in a mouse model with folic acid-induced CKD. Activated AMPK phosphorylates and inactivates ACC activity, which improves lipid availability for energy generation from fatty acids in renal tubular cells. By use of ACC knock-in (KI) mice, with inactivating Ser to Ala mutations preventing ACC phosphorylation, it was observed that metformin’s protective effects were abrogated, as folic acid induced tubulointerstitial fibrosis, and lipid accumulation were not attenuated. The antifibrotic effect of metformin is, thus; dependent on its ability to increase phosphorylation of ACC, which in turn increases fatty acid oxidation in damaged renal tissues [71]. In a rat model of CKD-mineral and bone disorder, Neven et al. [19] showed that daily metformin treatment (200 mg/kg/day p.o.) was able to strongly attenuate development of severe CKD, as evidenced by a preserved renal function with maintained mineral homeostasis, and a significantly reduced renal inflammation, cellular infiltration and fibrosis [19]. 

The potential role of metformin in protecting the kidney from a nephrotoxic insult was examined by Morales et al. [71]. They hypothesized that metformin treatment (100 mg/kg/day p.o.) protected the kidney from gentamicin-induced toxicity in rats, a model of acute renal failure, through a mitochondria-dependent pathway. Indeed, endogenous oxidative stress has a major role in the severity of gentamicin-induced acute renal failure, suggesting that the protective effect, afforded by metformin, could be mediated, at least in part, by enhanced antioxidant defenses, and by its ability to prevent gentamicin-induced lipid peroxidation [71]. In a mouse model of unilateral ureteral obstruction, Cavaglieri et al. [72] showed that metformin treatment (200 mg/kg/day p.o.), initiated one day before surgery, prevented or slowed down the onset of renal inflammation and fibrosis. This amelioration was accompanied by increased activity of AMPK [72]. Declèves et al. [18] investigated the effects of AMPK activity, using agonists of AMPK, among which included metformin (300 mg/kg/day p.o.), on autophagy and cell stress proteins in a model of kidney ischemia-reperfusion (IR). They showed that IR led to downregulation of AMPK and autophagy which consequently induced cellular changes. Additionally, they demonstrated that induction of the AMPK/autophagy axis by metformin provided beneficial cellular effects which offered a viable strategy in reducing kidney IR injury [18].

Conversely, Lieberthal et al. [73] demonstrated that inhibition of the mammalian target of rapamycin (mTOR) impaired renal recovery, assessed by GFR, after IR in rats, which appeared to be due, at least in part, to the inhibition of tubular cell regeneration [73,74]. The mTOR pathway is one of the downstream signaling pathways regulated by AMPK [75]. Consequently, metformin, as AMPK activator, is able to inhibit the mTOR pathway and cause a delay in renal regeneration and repair [73,76]. However, Lieberthal et al. [74] also proved that the regeneration of tubular cells and recovery of GFR after IR in rats, although delayed by the mTOR inhibitor, rapamycin, eventually occurred, despite continued administration of the latter inhibitor [74].

## 7. Metformin’s Renoprotection: Molecular Mechanisms

**Figure 3:**
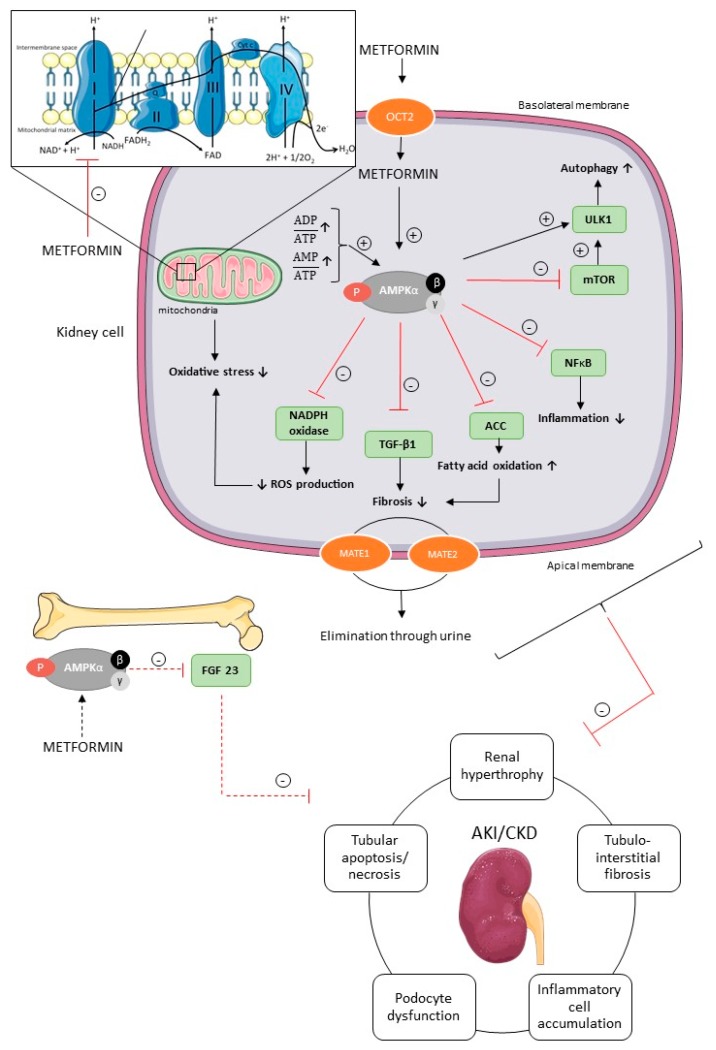
Potential underlying molecular mechanisms of metformin’s renoprotection. Metformin is transported into the renal tubular cells mainly through the plasma membrane transporter OCT2. Inside the renal cell metformin activates AMPK via two separate mechanisms, i.e., the inhibition of the mitochondrial respiratory chain complex 1, and subsequent increase in AMP/ATP and ADP/ATP ratio, and/or the direct activation of AMPK. AMPK has pleiotropic downstream signaling pathways involved in divergent cellular processes, such as autophagy, fatty acid oxidation, inflammation, fibrosis, oxidative stress, and reactive oxygen species (ROS) in renal cells and FGF23 production in bone cells, which have been shown to protect the kidney against AKI and CKD. Further, MATE1 and MATE2 contribute to the renal excretion of metformin. ACC, acetyl-CoA carboxylase; AKI, acute kidney injury; AMPK, AMP-activated protein kinase; CKD, chronic kidney disease; FGF23, fibroblast growth factor 23; MATE1, multidrug and toxin extrusion 1; MATE2, multidrug and toxin extrusion 2; mTOR, mammalian target of rapamycin; NADPH, nicotinamide adenine dinucleotide phosphate; NFκB, nuclear factor kappa B; OCT2, organic cation transporter 2; ROS, reactive oxygen species; TGF-β1, transforming growth factor β1; ULK1, uncoordinated-51 like kinase 1. Figure was produced using Servier Medical Art: https://smart.servier.com/ (accessed on 2 November 2018).

Knowledge about the molecular mechanism by which metformin appears to prevent or attenuate AKI and CKD is fragmentary, however, it is clear that metformin protects the kidney via pleiotropic actions against different aspects of the pathophysiology of renal diseases, as presented in Figure 3.

Metformin, a positively charged molecule at physiological pH, enters the renal epithelial cells through the organic cation transporter 2 (OCT2), which is predominantly expressed at the basolateral membrane in the renal tubules [1,2]. Multidrug and toxin extrusion 1 (MATE1) and multidrug and toxin extrusion 2 (MATE2) transporters contribute to the renal excretion of metformin, which is eliminated, unchanged, in the urine by glomerular filtration and tubular secretion [1,2]. Metformin activates AMPK via two separate mechanisms, i.e., direct activation of AMPK via phosphorylation of its activating loop at Thr-172 of the α-subunit, and indirectly by inhibiting the mitochondrial respiratory chain complex 1 [22,76,77]. Indeed, metformin acts on mitochondria by inhibiting complex 1 of the respiratory chain, which leads to a reduction in cellular energy charge [22,77]. When ATP has been extensively utilized, the energy-sensing kinase AMPK is activated, which acts to restore energy homeostasis by switching to catabolic pathways for the generation of ATP [4,78].

The activation of inflammatory cells and production of extracellular matrix is regulated by transforming growth factor β1 (TGF-β1) in the kidney [16,79]. Metformin (10 mM) is able to diminish TGF-β1 expression in mouse renal fibroblasts in vitro and, consequently, alleviate pro-fibrotic gene expression via AMPK activation, which in vivo eventually should reduce interstitial fibrosis, one of the key pathological events of CKD [16,79]. Additionally, the antifibrotic effect of metformin is, at least in part, dependent on the ability of metformin to increase fatty acid oxidation via the AMPK-ACC pathway, as discussed previously. Oxidative stress is also involved in the pathogenesis of AKI and CKD [80,81]. Reactive oxygen species (ROS), such as superoxide anion (O_2_^−^) and hydrogen peroxide (H_2_O_2_), normally drives the cellular responses for tissue repair processes, inflammation and defense mechanisms. In pathological situations, however, the overproduction of free radicals contributes to cell and tissue injury [80,81,82]. In patients with renal dysfunction, the balance between ROS production and antioxidative defense mechanisms is disturbed. Furthermore, oxidative stress seems to increase as CKD progresses [82,83]. In the kidney, the enzymatic complex nicotinamide adenine dinucleotide phosphate (NADPH) oxidase is involved in ROS production [84]. In vitro studies further revealed that metformin (2 mM) prevents oxidative stress in podocytes, and decreases ROS production by inhibiting NADPH oxidase [85]. Moreover, metformin (0.01–0.1 mM) is able to protect tubular cells and renal tissue against tissue damage caused by oxidative stress, at least in part through a mitochondria-dependent pathway, as mentioned previously [71,86]. Recent literature has put forward autophagy as a possible mechanism in the pathology of AKI and CKD [87,88,89,90]. Indeed, the autophagic flux, i.e., the formation of autophagosomes and their fusion with lysosomes, is crucial for cell survival and a growing body of evidence suggests that a deficient autophagic flux may contribute to a broad spectrum of diseases, including AKI and CKD [91]. The mammalian autophagy-initiating uncoordinated-51 like kinase 1 (ULK1) is regulated by AMPK and mTOR [92]. Activated AMPK inhibits mTOR, leading to ULK1–AMPK interaction and activation of ULK1, which induces autophagy [92]. Metformin, as an AMPK agonist, may thus advance or prolong an inherent autophagic response, which may play a protective role against AKI, and the development and progression of CKD [87,90].

Recently, fibroblast growth factor 23 (FGF23) has gained broad attention as a disease biomarker in CKD, since elevated FGF23 plasma concentrations are observed in an early stage of CKD, and increase proportionally with renal function decline [93]. FGF23 is a phosphaturic hormone responsible for phosphate handling and vitamin D metabolism, with the kidney as an important target [93,94]. Further, FGF23 may actively contribute to the progression of CKD [93,94]. Since AMPK has been demonstrated as an important regulator of FGF23 production, it is tempting to speculate that metformin, being an AMPK activator, is able to inhibit FGF23 production, which is favorable to prevent deterioration of renal function [94].

## 8. Conclusions

Taken together, metformin, beyond its glucose-lowering actions in T2DM, may be considered a promising renoprotective compound in various types of renal diseases. Additional preclinical and longitudinal clinical trials are required to explore whether metformin is able to slow down, or even arrest, the progression of early kidney injury and CKD. Most of the reported in vitro and in vivo evidence on the potential effects of metformin has been based on a broad range of concentrations hampering the translation of experimental observations to the clinical situation. Experimental designs often include metformin concentrations or doses that are higher than what can be achieved in CKD patients. Additional in vivo studies using lower metformin doses would undoubtedly improve translation from preclinical studies to clinical trials. Further fundamental research is necessary to elucidate the complex network of signaling pathways by which metformin exerts its renoprotective effects, as this is indispensable to identify potentially new therapeutic targets.

## Abbreviations

ACC	acetyl-CoA carboxylase
AKI	acute kidney injury
AMPK	AMP-activated protein kinase
ATP	adenosine triphosphate
CKD	chronic kidney injury
eGFR	estimated glomerular filtration rate
ESRD	end stage renal disease
FADH2	flavin adenine dinucleotide
FDA	Food and Drug Administration
FGF23	fibroblast growth factor 23
GFR	glomerular filtration rate
GLP-1	glucagon-like peptide 1
IR	ischemia-reperfusion
i.v.	intravenous
KDOQI	kidney disease outcomes quality initiative
LKB1	liver kinase B1
MALA	metformin-associated lactic acidosis
MATE1	multidrug and toxin extrusion 1
MATE2	multidrug and toxin extrusion 2
mTOR	mammalian target of rapamycin
NADH	nicotinamide adenine dinucleotide
NADPH	nicotinamide adenine dinucleotide phosphate
NFκB,	nuclear factor kappa B
NKF	national kidney foundation
OCT1	organic cation transporter 1
OCT2	organic cation transporter 2
p.o.	per os
ROS	reactive oxygen species
T2DM	type 2 diabetes mellitus
TGF-β2	transforming growth factor β2
ULK1	uncoordinated-51 like kinase 1

## References

[1] Gong L., Goswami S., Giacomini K.M., Altman R.B., Klein T.E. (2012). Metformin pathways: Pharmacokinetics and pharmacodynamics. Pharmacogenet. Genom..

[2] Graham G.G., Punt J., Arora M., Day R.O., Doogue M.P., Duong J.K., Furlong T.J., Greenfield J.R., Greenup L.C., Kirkpatrick C.M. (2011). Clinical Pharmacokinetics of Metformin. Clin. Pharmacokinet..

[3] Imam T.H. (2017). Changes in metformin use in chronic kidney disease. Clin. Kidney J..

[4] Rena G., Hardie D.G., Pearson E.R. (2017). The mechanisms of action of metformin. Diabetologia.

[5] Marshall S.M. (2017). 60 years of metformin use: A glance at the past and a look to the future. Diabetologia.

[6] Bailey C.J. (2017). Metformin: Historical overview. Diabetologia.

[7] Panchapakesan U., Pollock C. (2018). Drug repurposing in kidney disease. Kidney Int..

[8] McCreight L.J., Bailey C.J., Pearson E.R. (2016). Metformin and the gastrointestinal tract. Diabetologia.

[9] Bahne E., Hansen M., Bronden A., Sonne D.P., Vilsboll T., Knop F.K. (2016). Involvement of glucagon-like peptide-1 in the glucose-lowering effect of metformin. Diabetes Obes. Metab..

[10] Wu H., Esteve E., Tremaroli V., Khan M.T., Caesar R., Mannerås-Holm L., Ståhlman M., Olsson L.M., Serino M., Planas-Fèlix M. (2017). Metformin alters the gut microbiome of individuals with treatment-naive type 2 diabetes, contributing to the therapeutic effects of the drug. Nat. Med..

[11] Lashen H. (2010). Role of metformin in the management of polycystic ovary syndrome. Ther. Adv. Endocrinol. Metab..

[12] Zi F., Zi H., Li Y., He J., Shi Q., Cai Z. (2018). Metformin and cancer: An existing drug for cancer prevention and therapy. Oncol. Lett..

[13] Nesti L., Natali A. (2017). Metformin effects on the heart and the cardiovascular system: A review of experimental and clinical data. Nutr. Metab. Cardiovasc. Dis..

[14] Li Y.A.N., Liu L.E.I., Wang B.I.N., Wang J.U.N., Chen D. (2013). Metformin in non-alcoholic fatty liver disease: A systematic review and meta-analysis. Biomed. Rep..

[15] Ibanez L., Ong K., Valls C., Marcos M.V., Dunger D.B., de Zegher F. (2006). Metformin treatment to prevent early puberty in girls with precocious pubarche. J. Clin. Endocrinol. Metab..

[16] Wang M., Weng X., Guo J., Chen Z., Jiang G., Liu X. (2016). Metformin alleviated EMT and fibrosis after renal ischemia-reperfusion injury in rats. Renal Fail..

[17] Li J., Gui Y., Ren J., Liu X., Feng Y., Zeng Z., He W., Yang J., Dai C. (2016). Metformin Protects against Cisplatin-Induced Tubular Cell Apoptosis and Acute Kidney Injury via AMPKalpha-regulated Autophagy Induction. Sci. Rep..

[18] Decleves A.E., Sharma K., Satriano J. (2014). Beneficial Effects of AMP-Activated Protein Kinase Agonists in Kidney Ischemia-Reperfusion: Autophagy and Cellular Stress Markers. Nephron Exp. Nephrol..

[19] Neven E., Vervaet B., Brand K., Gottwald-Hostalek U., Opdebeeck B., De Mare A., Verhulst A., Lalau J.D., Kamel S., De Broe M.E. (2018). Metformin prevents the development of severe chronic kidney disease and its associated mineral and bone disorder. Kidney Int..

[20] McCommis K.S., Finck B.N. (2015). Mitochondrial pyruvate transport: A historical perspective and future research directions. Biochem. J..

[21] Cool B., Zinker B., Chiou W., Kifle L., Cao N., Perham M., Dickinson R., Adler A., Gagne G., Iyengar R. (2006). Identification and characterization of a small molecule AMPK activator that treats key components of type 2 diabetes and the metabolic syndrome. Cell Metab..

[22] Owen M.R., Doran E., Halestrap A.P. (2000). Evidence that metformin exerts its anti-diabetic effects through inhibition of complex 1 of the mitochondrial respiratory chain. Biochem. J..

[23] Wilcock C., Bailey C.J. (1994). Accumulation of metformin by tissues of the normal and diabetic mouse. Xenobiotica.

[24] He L., Wondisford F.E. (2015). Metformin Action: Concentrations Matter. Cell Metab..

[25] Shaw R.J., Lamia K.A., Vasquez D., Koo S.-H., Bardeesy N., DePinho R.A., Montminy M., Cantley L.C. (2005). The Kinase LKB1 Mediates Glucose Homeostasis in Liver and Therapeutic Effects of Metformin. Science.

[26] Cao J., Meng S., Chang E., Beckwith-Fickas K., Xiong L., Cole R.N., Radovick S., Wondisford F.E., He L. (2014). Low concentrations of metformin suppress glucose production in hepatocytes through AMP-activated protein kinase (AMPK). J. Biol. Chem..

[27] Foretz M., Hébrard S., Leclerc J., Zarrinpashneh E., Soty M., Mithieux G., Sakamoto K., Andreelli F., Viollet B. (2010). Metformin inhibits hepatic gluconeogenesis in mice independently of the LKB1/AMPK pathway via a decrease in hepatic energy state. J. Clin. Investig..

[28] Miller R.A., Chu Q., Xie J., Foretz M., Viollet B., Birnbaum M.J. (2013). Biguanides suppress hepatic glucagon signalling by decreasing production of cyclic AMP. Nature.

[29] Madiraju A.K., Erion D.M., Rahimi Y., Zhang X.M., Braddock D.T., Albright R.A., Prigaro B.J., Wood J.L., Bhanot S., MacDonald M.J. (2014). Metformin suppresses gluconeogenesis by inhibiting mitochondrial glycerophosphate dehydrogenase. Nature.

[30] Hunter R.W., Hughey C.C., Lantier L., Sundelin E.I., Peggie M., Zeqiraj E., Sicheri F., Jessen N., Wasserman D.H., Sakamoto K. (2018). Metformin reduces liver glucose production by inhibition of fructose-1-6-bisphosphatase. Nat. Med..

[31] Bellomo R., Kellum J.A., Ronco C. (2012). Acute kidney injury. Lancet.

[32] Chawla L.S., Kimmel P.L. (2012). Acute kidney injury and chronic kidney disease: An integrated clinical syndrome. Kidney Int..

[33] Ferenbach D.A., Bonventre J.V. (2015). Mechanisms of maladaptive repair after AKI leading to accelerated kidney ageing and CKD. Nat. Rev. Nephrol..

[34] Kidney Disease: Improving Global Outcomes (KDIGO) CKD Work Group (2012). KDIGO 2012 clinical practice guideline for the evaluation and management of chronic kidney disease. Kidney Int. Suppl..

[35] Thadhani R., Pascual M., Bonventre J.V. (1996). Acute renal failure. N. Engl. J. Med..

[36] Basile D.P., Anderson M.D., Sutton T.A. (2012). Pathophysiology of Acute Kidney Injury. Compr. Physiol..

[37] Lameire N., Van Biesen W., Vanholder R. (2005). Acute renal failure. Lancet.

[38] Humphreys B.D., Cantaluppi V., Portilla D., Singbartl K., Yang L., Rosner M.H., Kellum J.A., Ronco C. (2016). Targeting Endogenous Repair Pathways after AKI. J. Am. Soc. Nephrol..

[39] Coca S.G., Singanamala S., Parikh C.R. (2012). Chronic kidney disease after acute kidney injury: A systematic review and meta-analysis. Kidney Int..

[40] Jha V., Garcia-Garcia G., Iseki K., Li Z., Naicker S., Plattner B., Saran R., Wang A.Y., Yang C.W. (2013). Chronic kidney disease: Global dimension and perspectives. Lancet.

[41] De Broe M.E., Gharbi M.B., Elseviers M. (2016). Maremar, prevalence of chronic kidney disease, how to avoid over-diagnosis and under-diagnosis. Néphrol. Thér..

[42] Kidney Disease: Improving Global Outcomes (KDIGO) CKD Work Group (2013). KDIGO 2012 Clinical practice guideline for the evaluation and management of chronic kidney disease. Kidney Int. Suppl..

[43] Lopez-Giacoman S., Madero M. (2015). Biomarkers in chronic kidney disease, from kidney function to kidney damage. World J. Nephrol..

[44] Webster A.C., Nagler E.V., Morton R.L., Masson P. (2017). Chronic Kidney Disease. Lancet.

[45] Schlondorff D.O. (2008). Overview of factors contributing to the pathophysiology of progressive renal disease. Kidney Int..

[46] Levey A.S., Becker C., Inker L.A. (2015). Glomerular Filtration Rate and Albuminuria for Detection and Staging of Acute and Chronic Kidney Disease in Adults: A Systematic Review. JAMA.

[47] (2002). K/DOQI clinical practice guidelines for chronic kidney disease: Evaluation, classification, and stratification. Am. J. Kidney Dis..

[48] Luyckx V.A., Tuttle K.R., Garcia-Garcia G., Gharbi M.B., Heerspink H.J.L., Johnson D.W., Liu Z.-H., Massy Z.A., Moe O., Nelson R.G. (2017). Reducing major risk factors for chronic kidney disease. Kidney Int. Suppl..

[49] Lewis E.J., Hunsicker L.G., Clarke W.R., Berl T., Pohl M.A., Lewis J.B., Ritz E., Atkins R.C., Rohde R., Raz I. (2001). Renoprotective effect of the angiotensin-receptor antagonist irbesartan in patients with nephropathy due to type 2 diabetes. N. Engl. J. Med..

[50] Brenner B.M., Cooper M.E., de Zeeuw D., Keane W.F., Mitch W.E., Parving H.-H., Remuzzi G., Snapinn S.M., Zhang Z., Shahinfar S. (2001). Effects of Losartan on Renal and Cardiovascular Outcomes in Patients with Type 2 Diabetes and Nephropathy. N. Engl. J. Med..

[51] Lalau J.D., Arnouts P., Sharif A., De Broe M.E. (2015). Metformin and other antidiabetic agents in renal failure patients. Kidney Int..

[52] Chan N.N., Brain H.P., Feher M.D. (1999). Metformin-associated lactic acidosis: A rare or very rare clinical entity?. Diabet. Med..

[53] Fall P.J., Szerlip H.M. (2005). Lactic Acidosis: From Sour Milk to Septic Shock. J. Intensive Care Med..

[54] DeFronzo R., Fleming G.A., Chen K., Bicsak T.A. (2016). Metformin-associated lactic acidosis: Current perspectives on causes and risk. Metab. Clin. Exp..

[55] Lipska K.J. (2017). Metformin Use in Patients With Historical Contraindications. Ann. Intern. Md..

[56] Misbin R.I., Green L., Stadel B.V., Gueriguian J.L., Gubbi A., Fleming G.A. (1998). Lactic Acidosis in Patients with Diabetes Treated with Metformin. N. Engl. J. Med..

[57] Van Berlo-van de Laar I.R., Vermeij C.G., Doorenbos C.J. (2011). Metformin associated lactic acidosis: Incidence and clinical correlation with metformin serum concentration measurements. J. Clin. Pharm. Ther..

[58] Kajbaf F., Lalau J.-D. (2013). The prognostic value of blood pH and lactate and metformin concentrations in severe metformin-associated lactic acidosis. BMC Pharmacol. Toxicol..

[59] Ekström N., Schiöler L., Svensson A.-M., Eeg-Olofsson K., Miao Jonasson J., Zethelius B., Cederholm J., Eliasson B., Gudbjörnsdottir S. (2012). Effectiveness and safety of metformin in 51 675 patients with type 2 diabetes and different levels of renal function: a cohort study from the Swedish National Diabetes Register. BMJ Open.

[60] Richy F.F., Sabidó-Espin M., Guedes S., Corvino F.A., Gottwald-Hostalek U. (2014). Incidence of Lactic Acidosis in Patients With Type 2 Diabetes With and Without Renal Impairment Treated With Metformin: A Retrospective Cohort Study. Diabetes Care.

[61] Eppenga W.L., Lalmohamed A., Geerts A.F., Derijks H.J., Wensing M., Egberts A., De Smet P.A.G.M., de Vries F. (2014). Risk of Lactic Acidosis or Elevated Lactate Concentrations in Metformin Users With Renal Impairment: A Population-Based Cohort Study. Diabetes Care.

[62] Crowley M.J., Diamantidis C.J., McDuffie J.R. Metformin Use in Patients with Historical Contraindications or Precautions [Internet]. https://www.ncbi.nlm.nih.gov/books/NBK409379/.

[63] Lalau J.D., Kajbaf F., Bennis Y., Hurtel-Lemaire A.S., Belpaire F., De Broe M.E. (2018). Metformin Treatment in Patients With Type 2 Diabetes and Chronic Kidney Disease Stages 3A, 3B, or 4. Diabetes Care.

[64] Bell S., Farran B., McGurnaghan S., McCrimmon R.J., Leese G.P., Petrie J.R., McKeigue P., Sattar N., Wild S., McKnight J. (2017). Risk of acute kidney injury and survival in patients treated with Metformin: An observational cohort study. BMC Nephrol..

[65] Stephen J., Anderson-Haag T.L., Gustafson S., Snyder J.J., Kasiske B.L., Israni A.K. (2014). Metformin use in kidney transplant recipients in the United States: An observational study. Am. J. Nephrol..

[66] Hippisley-Cox J., Coupland C. (2016). Diabetes treatments and risk of amputation, blindness, severe kidney failure, hyperglycaemia, and hypoglycaemia: Open cohort study in primary care. BMJ.

[67] Crowley M.J., Diamantidis C.J., McDuffie J.R., Cameron C.B., Stanifer J.W., Mock C.K., Wang X., Tang S., Nagi A., Kosinski A.S. (2017). Clinical Outcomes of Metformin Use in Populations With Chronic Kidney Disease, Congestive Heart Failure, or Chronic Liver Disease: A Systematic Review. Ann. Intern. Med..

[68] Hsu W.H., Hsiao P.J., Lin P.C., Chen S.C., Lee M.Y., Shin S.J. (2018). Effect of metformin on kidney function in patients with type 2 diabetes mellitus and moderate chronic kidney disease. Oncotarget.

[69] Satriano J., Sharma K., Blantz R.C., Deng A. (2013). Induction of AMPK activity corrects early pathophysiological alterations in the subtotal nephrectomy model of chronic kidney disease. Am. J. Physiol. Renal Physiol..

[70] Lee M., Katerelos M., Gleich K., Galic S., Kemp B.E., Mount P.F., Power D.A. (2018). Phosphorylation of Acetyl-CoA Carboxylase by AMPK Reduces Renal Fibrosis and Is Essential for the Anti-Fibrotic Effect of Metformin. J. Am. Soc. Nephrol..

[71] Morales A.I., Detaille D., Prieto M., Puente A., Briones E., Arévalo M., Leverve X., López-Novoa J.M., El-Mir M.-Y. (2010). Metformin prevents experimental gentamicin-induced nephropathy by a mitochondria-dependent pathway. Kidney Int..

[72] Cavaglieri R.C., Day R.T., Feliers D., Abboud H.E. (2015). Metformin prevents renal interstitial fibrosis in mice with unilateral ureteral obstruction. Mol. Cell. Endocrinol..

[73] Lieberthal W., Fuhro R., Andry C.C., Rennke H., Abernathy V.E., Koh J.S., Valeri R., Levine J.S. (2001). Rapamycin impairs recovery from acute renal failure: Role of cell-cycle arrest and apoptosis of tubular cells. Am. J. Physiol. Renal Physiol..

[74] Lieberthal W., Fuhro R., Andry C., Patel V., Levine J.S. (2006). Rapamycin delays but does not prevent recovery from acute renal failure: Role of acquired tubular resistance. Transplantation.

[75] Gwinn D.M., Shaw R.J., Tamanoi F., Hall M.N. (2010). 3—AMPK Control of mTOR Signaling and Growth. The Enzymes.

[76] Hardie D.G., Schaffer B.E., Brunet A. (2016). AMPK: An Energy-Sensing Pathway with Multiple Inputs and Outputs. Trends Cell Biol..

[77] Andrzejewski S., Gravel S.-P., Pollak M., St-Pierre J. (2014). Metformin directly acts on mitochondria to alter cellular bioenergetics. Cancer Metab..

[78] Mihaylova M.M., Shaw R.J. (2011). The AMP-activated protein kinase (AMPK) signaling pathway coordinates cell growth, autophagy, & metabolism. Nat. Cell Biol..

[79] Lu J., Shi J., Li M., Gui B., Fu R., Yao G., Duan Z., Lv Z., Yang Y., Chen Z. (2015). Activation of AMPK by metformin inhibits TGF-beta-induced collagen production in mouse renal fibroblasts. Life Sci..

[80] Modaresi A., Nafar M., Sahraei Z. (2015). Oxidative stress in chronic kidney disease. Iran. J. Kidney Dis..

[81] Pavlakou P., Liakopoulos V., Eleftheriadis T., Mitsis M., Dounousi E. (2017). Oxidative Stress and Acute Kidney Injury in Critical Illness: Pathophysiologic Mechanisms—Biomarkers—Interventions, and Future Perspectives. Oxid. Med. Cell. Longev..

[82] Kao M.P.C., Ang D.S.C., Pall A., Struthers A.D. (2009). Oxidative stress in renal dysfunction: Mechanisms, clinical sequelae and therapeutic options. J. Hum. Hypertens..

[83] Signorini L., Granata S., Lupo A., Zaza G. (2017). Naturally Occurring Compounds: New Potential Weapons against Oxidative Stress in Chronic Kidney Disease. Int. J. Mol. Sci..

[84] Sedeek M., Nasrallah R., Touyz R.M., Hébert R.L. (2013). NADPH Oxidases, Reactive Oxygen Species, and the Kidney: Friend and Foe. J. Am. Soc. Nephrol..

[85] Piwkowska A., Rogacka D., Jankowski M., Dominiczak M.H., Stepinski J.K., Angielski S. (2010). Metformin induces suppression of NAD(P)H oxidase activity in podocytes. Biochem. Biophys. Res. Commun..

[86] Ishibashi Y., Matsui T., Takeuchi M., Yamagishi S. (2012). Metformin inhibits advanced glycation end products (AGEs)-induced renal tubular cell injury by suppressing reactive oxygen species generation via reducing receptor for AGEs (RAGE) expression. Horm. Metab. Res..

[87] He L., Livingston M.J., Dong Z. (2014). Autophagy in Acute Kidney Injury and Repair. Nephron Clin. Pract..

[88] Huber T.B., Edelstein C.L., Hartleben B., Inoki K., Jiang M., Koya D., Kume S., Lieberthal W., Pallet N., Quiroga A. (2012). Emerging role of autophagy in kidney function, diseases and aging. Autophagy.

[89] Ding Y., Kim S., Lee S.Y., Koo J.K., Wang Z., Choi M.E. (2014). Autophagy regulates TGF-beta expression and suppresses kidney fibrosis induced by unilateral ureteral obstruction. J. Am. Soc. Nephrol..

[90] Mao S., Zhang J. (2015). Role of autophagy in chronic kidney diseases. Int. J. Clin. Exp. Med..

[91] Liu N., Shi Y., Zhuang S. (2016). Autophagy in Chronic Kidney Diseases. Kidney Dis..

[92] Kim J., Kundu M., Viollet B., Guan K.-L. (2011). AMPK and mTOR regulate autophagy through direct phosphorylation of Ulk1. Nat. Cell Biol..

[93] Wahl P., Wolf M. (2012). FGF23 in chronic kidney disease. Adv. Exp. Med. Biol..

[94] Glosse P., Feger M., Mutig K., Chen H., Hirche F., Hasan A.A., Gaballa M.M.S., Hocher B., Lang F., Foller M. (2018). AMP-activated kinase is a regulator of fibroblast growth factor 23 production. Kidney Int..

